# Subtractive, divisive and non-monotonic gain control in feedforward nets linearized by noise and delays

**DOI:** 10.3389/fncom.2014.00019

**Published:** 2014-02-25

**Authors:** Jorge F. Mejias, Alexandre Payeur, Erik Selin, Leonard Maler, André Longtin

**Affiliations:** ^1^Department of Physics, University of OttawaOttawa, ON, Canada; ^2^Center for Neural Science, New York UniversityNew York, NY, USA; ^3^Department of Cell and Molecular Medicine, University of OttawaOttawa, ON, Canada

**Keywords:** gain control, feedforward network, subtractive, divisive, non-monotonic, linearization by delay, weakly electric fish

## Abstract

The control of input-to-output mappings, or gain control, is one of the main strategies used by neural networks for the processing and gating of information. Using a spiking neural network model, we studied the gain control induced by a form of inhibitory feedforward circuitry—also known as “open-loop feedback”—, which has been experimentally observed in a cerebellum-like structure in weakly electric fish. We found, both analytically and numerically, that this network displays three different regimes of gain control: subtractive, divisive, and non-monotonic. Subtractive gain control was obtained when noise is very low in the network. Also, it was possible to change from divisive to non-monotonic gain control by simply modulating the strength of the feedforward inhibition, which may be achieved via long-term synaptic plasticity. The particular case of divisive gain control has been previously observed *in vivo* in weakly electric fish. These gain control regimes were robust to the presence of temporal delays in the inhibitory feedforward pathway, which were found to linearize the input-to-output mappings (or f-I curves) via a novel variability-increasing mechanism. Our findings highlight the feedforward-induced gain control analyzed here as a highly versatile mechanism of information gating in the brain.

## 1. Introduction

The mapping between the input arriving to a neuron and its evoked firing rate has constituted one of the major interests in the study of neural systems over the last decades (Perkel et al., 1964; Segundo, 1970; Salinas and Sejnowski, 2001). In many situations, neurons are able to perform a scaling operation on their response to input. For instance, contrast invariance of signal cancelation (Mejias et al., 2013) and object representation (Serrano et al., 2013), receptive field properties (Alitto and Usrey, 2004), and orientation selectivity (Ferster and Miller, 2000) require a contrast-dependent scaling of responses in sensory areas. Gaze direction also scales the spiking response rate of neurons in the primary visual (Trotter and Celebrini, 1999) and posterior parietal (Andersen and Mountcastle, 1983) cortices. Scaling can also be context-specific, as found in the auditory pathway of crickets (Hildebrandt et al., 2011). Finally, it is known that cortical circuits are able to modulate their response gain depending on the input frequency (Abbott et al., 1997; Tsodyks and Markram, 1997; Rothman et al., 2009) by means of short-term synaptic plasticity.

The scaling and control of input–output behavior of neural systems is often characterized by the so-called f-I curve, which displays the output firing rate versus input current to the neuron. In particular, the slope of such a dependency, or *gain*, constitutes a useful indicator of the behavior of the neuron. If the gain of the f-I curve is high, a small change in the input current will be mapped by the cell into a large change in the output firing rate, increasing the sensitivity of the neuron to weak stimuli. On the other hand, a low gain of the f-I curve translates large changes in the input current to small changes in the output firing rate, and this allows the neuron to encode a broad range of stimulus intensities into a physiologically plausible range of firing rates. This framework is strictly valid only when the stimulus evolves slowly compared to the integration time scale of the neuron, although the applicability of this formalism to fast varying stimuli has also been considered (Ly and Doiron, 2009).

**Figure 1:**
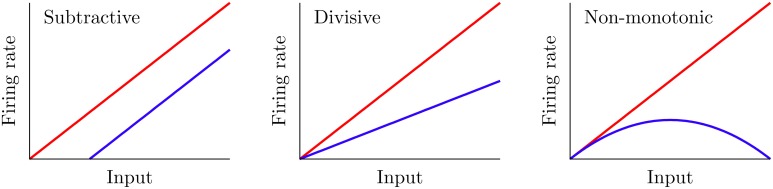
**Three different gain control effects commonly observed in real neural systems**. The red curve corresponds to the f-I curve before the gain modulation, and the blue curve shows it after the modulation. Subtractive gain control implies a shift of the original f-I curve, while divisive control leads to a change in slope. Non-monotonic gain control usually reflects more sophisticated input–output properties of neurons and synapses.

Certain mechanisms allow a neuron to modify its f-I curve, a phenomenon which is known as gain control. Figure 1 shows three possible examples of gain control on the behavior of a neuron. A simple gain control effect that one could think of is a shift-like effect known in the literature as subtractive gain control. In this case, the f-I curve experiences a subtractive (or additive) shift toward larger (or smaller) values of the input current without varying its overall shape. A large number of mechanisms, such as the introduction of some level of shunting inhibition (Holt and Koch, 1997), are able to produce this form of gain control.

Mechanisms providing the other two types of gain control shown in Figure 1 have been more elusive. Divisive gain control is often assumed in rate models of neurons and neural populations (Carandini and Heeger, 1994; Chance and Abbott, 2000). However, biophysical mechanisms for such a gain modulation have been hard to identify (Holt and Koch, 1997; Doiron et al., 2001; Chance et al., 2002). On the other hand, non-monotonic gain control has been reported to be induced by short-term depression (de la Rocha and Parga, 2005; Lewis et al., 2007). However, general biophysical mechanisms which cover all these gain control strategies have been difficult to identify and characterize up to date.

In this work, we present a computational model of a neural circuit which is able to display these three types of gain control (subtractive, divisive, and non-monotonic). We consider a generic neural circuit in which neurons receive a given stimulus both directly, i.e., from sensory receptors, and indirectly, via inhibitory interneurons driven by the same stimulus (a pathway referred here as feedforward inhibition, but that is also known in this context as *open-loop feedback* Litwin-Kumar et al., 2012). Our results, both numerical and analytical, show that these neurons can exhibit the three different types of gain control described above (and shown in Figure 1). The particular type of gain control exhibited by the system depends on (1) the noise level present on neurons and (2) the strength of the negative input provided by the inhibitory neurons, which may be easily modulated in real circuits via long-term synaptic plasticity and therefore provides a highly versatile gain control mechanism. We also carefully analyze the conditions under which the f-I curve of the neural system becomes non-monotonic.

**Figure 2:**
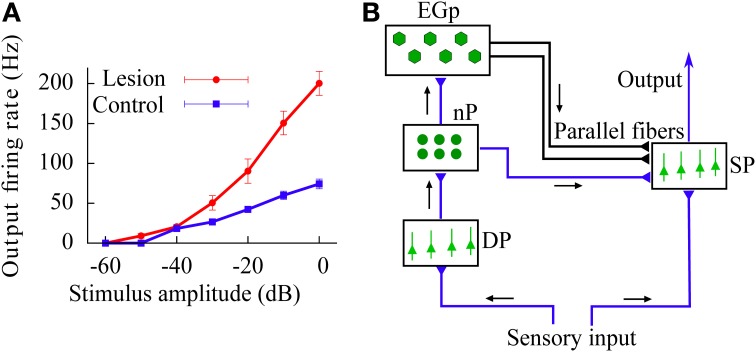
**(A)** *In vivo* experimental recording of the firing rate (relative to baseline activity) of SP neurons in the ELL of the weakly electric fish, as a function of the stimulus intensity. Data from control fish is compared with data from fish for which synapses providing the feedforward inhibition via the cerebellar parallel fibers have been removed (lesion). This implies that the effect caused by the parallel fibers is mainly a divisive gain control. Data taken from Bastian (1986b). **(B)** Scheme of the neural circuit considered in our study. Black arrows show the direction of the input transmission. Parallel fibers are represented by the black lines drawn from the EGp to the SP population. Note that both SP and DP cells receive the sensory input.

In addition to these results, we also study the above gain control mechanism in the case in which the feedforward inhibition introduces a temporal delay in the signal transmission (reflecting the presence of finite propagation times in the real brain). The existence of such a delay increases the variability of the effective current arriving to the neurons, which in turn leads to a linearization of the f-I curves. The gain control induced by feedforward inhibition is also present in this more realistic scenario. Finally, we use our model to explain the divisive gain control observed *in vivo* in the superficial pyramidal (SP) neurons of the electrosensory lateral-line lobe (ELL), a cerebellum-like structure of the weakly electric fish *Apteronotus leptorhynchus* (Bastian, 1986b; see also Figure 2A).

## 2. Materials and methods

A simplified scheme of the cerebellum-like circuit that we consider in this study is shown in Figure 2B. This circuit resembles the electrosensory lateral-line lobe (ELL), a primary sensory area in the brain of the weakly electric fish *Apteronotus leptorhynchus* (Maler et al., 1991), although our results can be easily generalized to any circuit in the brain presenting a degree of feedforward inhibition comparable to the one present in the circuit of Figure 2B.

Briefly, sensory inputs coming from electroreceptors arrive to two different pools of neurons: the superficial pyramidal (SP) and the deep pyramidal (DP) neurons (Maler, 2007). Both populations are mainly feedforward, and neurons within a population do not connect between themselves (i.e., no recurrent connectivity). From the population of DP neurons, the stimulus is transmitted to the nucleus praeminentialis (nP) and then to a population of granule cells called the eminentia granularis (EGp). A subset of neurons in the nP directly connects to SP cells and provides an inhibitory feedforward signal. Granule cells of the EGp transmit the signal they receive from a subgroup of nP cells to the SP cells via a massive set of parallel fibers (Berman and Maler, 1999). Apart from a direct excitatory synaptic connection with SP cells, the parallel fibers also synapse onto inhibitory interneurons which then project onto the soma and/or apical dendrites of SP cells and strongly inhibit them. As a consequence of this strong inhibition (or, more precisely, disynaptic inhibition), the overall contribution of parallel fibers to SP neurons is mainly inhibitory (Bastian, 1986b). Note that another subset of nP neurons is excitatory and connects directly to the SP cells as well as disynaptically through inhibitory interneurons. A compelling option is to amalgamate the latter pathway with the other one going directly from the nP to the SP cells. We shall make no further reference to this third pathway. The pathway followed by the sensory stimulus going from DP neurons to SP neurons via the nP and the EGp is called open-loop feedback, or feedforward inhibition (note that both populations receive the stimuli, and thus lie at the same processing stage, however, DP cells send signals to SP cells but do not receive them back). Finally, neurons in the SP population project onto higher areas of the brain.

We consider a population of *N* deep pyramidal (DP) neurons receiving sensory input from electroreceptors. The membrane potential *V*^*D*^_*i*_(*t*) of the DP neuron *i* follows a simple leaky integrate-and-fire (LIF) dynamics,

(1) $$\tau_{m}\frac{dV_{i}^{D}(t)}{dt}=-V_{i}^{D}(t)+\mu +\sqrt{\tau_{m}}\sigma \xi_{i}^{D}(t),$$

where τ_*m*_ is the membrane time constant, μ is the sensory input (that we consider constant for simplicity), and ξ^*D*^_*i*_(*t*) is a Gaussian white noise of zero mean and delta-type autocorrelation 〈ξ^*D*^_*i*_(*t*)ξ^*D*^_*j*_(*t* + τ)〉 = δ_*ij*_δ(τ), with δ_*ij*_ and δ(*t*) being the Kronecker and Dirac delta, respectively. This noise term was included in the model to reflect the intrinsic stochasticity of the deep neurons (Bastian and Nguyenkim, 2001). The factor σ reflects the noise intensity of this stochastic term.

Following the typical dynamics of the LIF model, when the membrane potential reaches a certain threshold *V*_*th*_, an action potential is generated by the neuron and the membrane potential is reset to *V*_*r*_, and remains there for a refractory period τ_*r*_.

In the real neural circuit, the population of DP neurons projects onto the nP, then some nP cells project directly onto SP cells while others connect with the EGp, whose granule cells make synaptic contact with the SP neurons both directly and through disynaptic inhibition. In our model, we simplify these intricate feedforward connections by assuming that the activity of the DP population ultimately drives the dynamics of the SP cells, so that the input that a SP neuron receives from the feedforward inhibition pathway is given by

(2) $$f(t)=\tau_{m}G\frac{1}{N}\sum_{i=1}^{N}\sum_{t_{i,k}}s\text{​}\left(t-t_{i,k}\right)\text{​}.$$

Here, the first sum runs over all DP neurons, and the second sum runs over the spike times of each presynaptic DP neuron (i.e., *t*_*i,k*_ is the *k*-th spike time from the *i*-th presynaptic neuron). The factor *G*/*N* may be identified as the effective strength of synapses onto the SP cells. Since the net polarity of the DP to SP feedforward pathway seems mainly inhibitory (Bastian, 1986b; Doiron et al., 2003), we assume *G* ≤ 0.

The synaptic filter function, *s*(τ), describes the effect of a given presynaptic spike on the postsynaptic potential, and it is given by a delayed alpha function

(3) $$s\left(\tau \right)=\left(\frac{\tau -\tau_{d}}{\tau_{s}^{2}}\right)e^{-\frac{\tau -\tau_{d}}{\tau_{s}}}\Theta \text{​}\left(\tau -\tau_{d}\right)\text{​},$$

where τ_*s*_ is the synaptic transmission time scale, τ_*d*_ is a temporal delay, and Θ(*x*) is the Heaviside step function (i.e., Θ(*x*) = 1 if *x* > 0, and Θ(*x*) = 0 otherwise). Note that *s*(τ) is normalized so that integration over a large enough time window yields unity.

Equation (2) can also be written by using a convolution of the population average for the DP cells and the synaptic filter. The population average is

(4) $$Y^{D}(t)=\frac{1}{N}\sum_{i=1}^{N}y_{i}^{D}(t),$$

where *y*^*D*^_*i*_(*t*) = ∑_*t*_*i,k*__ δ(*t* − *t*_*i,k*_) is the spike train of DP neuron *i*. Hence,

(5) $$f(t)=\tau_{m}G\left(s*Y^{D}\right)(t),$$

where (*a* ∗ *b*)(*t*) is the convolution of functions *a*(*t*) and *b*(*t*).

Since all SP neurons will receive, on average, the same input from sensory receptors and from the feedforward inhibitory pathway, we will consider the response of a typical SP cell, and the output statistics will be valid for all the other SP neurons. The membrane potential *V*^*S*^(*t*) of a typical SP neuron is described, as in the case of DP neurons, by a simple leaky integrate-and-fire (LIF) dynamics,

(6) $$\tau_{m}\frac{dV^{S}(t)}{dt}=-V^{S}(t)+\mu +f(t)+\sqrt{\tau_{m}}\sigma \xi^{S}(t),$$

where, as for the DP neurons, μ is the sensory input and the last term considers the intrinsic stochasticity of the neuron (with ξ^*S*^(*t*) being a Gaussian white noise of zero mean and delta-type autocorrelation). The term *f*(*t*) is the input coming from the DP cells and is already known. Again, when the membrane potential reaches the threshold *V*_*th*_, a spike is generated and *V*^*S*^(*t*) is reset to *V*_*r*_ during a period τ_*r*_.

Unless specified otherwise, we choose the following values for the time constants: τ_*m*_ = 10 ms, τ_*r*_ = 1 ms, τ_*s*_ = 5 ms, and τ_*d*_ = 10 ms. For parameters related to the membrane potential we consider, without loss of generality, dimensionless units. We set *V*_*th*_ = 1 and *V*_*r*_ = 0, and therefore μ, σ, and *G* will be in “resting-to-threshold” units. For simulations, we used a DP population of *N* = 500 neurons unless another size is specified.

## 3. Results

In the following sections, we will analyze the gain control performed by the DP population on the SP neuron. Even though the gain is properly defined as the derivative of a f-I curve, we shall present f-I curves only since these are the ones usually obtained experimentally—and therefore conclusions arising from the analysis of these curves would naturally translate into results for the derivative of f-I curves. We will compare the f-I curve of a SP cell with and without the feedforward modulation, for different parameter regimes. From Equation (5), setting *G* = 0 makes the feedforward signal *f*(*t*) vanish. But with *G* = 0, the DP and SP neurons share the same f-I curve since they share the same properties (compare Equation 6 with *f*(*t*) = 0 and Equation 1). Hence, the gain control can be fully understood by comparing the SP cell's f-I curve with that of a DP neuron. A gain control will be called subtractive when there is a noticeable shift along the input (i.e., μ) axis in the SP f-I curve with respect to the DP f-I curve. Divisive gain control occurs when the slope (derivative) of the SP f-I curve is mainly scaled by a constant factor with respect to that of the DP population. Finally, a non-monotonic gain control involves the presence of a maximum in the SP f-I curve, so that the scaling factor connecting the slopes of the SP and DP f-I curves changes sign with the input (see Figure 1).

### 3.1. Subtractive gain control

We start our analysis by considering the limit case in which neurons in our system are deterministic (i.e., σ = 0). In this case, the mean firing rate of the DP neurons may be easily obtained by solving Equation (1) for a given neuron. Since the input is the same for all DP neurons, their firing rate will also be the same. One obtains (Tuckwell, 1988)

(7) $$r_{D}={\left[\tau_{r}+\tau_{m}\log\left(\frac{V_{r}-\mu }{V_{th}-\mu }\right)\right]}^{-1}\Theta \left(\mu -V_{th}\right)\text{​}.$$

For convenience, we define the *effective bias* to the SP cell as the sum of the sensory input plus the feedfoward inhibitory contribution, i.e., μ_eff_ = μ + *f*(*t*). In the diffusion limit (Brunel, 2000), and ignoring fluctuation terms, the effective bias can be written as

(8) $$\mu_{\text{eff}}=\mu +\tau_{m}Gr_{D}.$$

In the case of a deterministic system, it takes the form

(9) $$\mu_{\text{eff}}=\mu +\frac{\tau_{m}G\;\Theta \text{​}\left(\mu -V_{th}\right)}{\tau_{r}+\tau_{m}\log\left(\frac{V_{r}-\mu }{V_{th}-\mu }\right)}\text{​}.$$

Finally, the expression for the SP firing rate is given by

(10) $$r_{S}={\left[\tau_{r}+\tau_{m}\log\left(\frac{V_{r}-\mu_{\text{eff}}}{V_{th}-\mu_{\text{eff}}}\right)\right]}^{-1}\Theta \text{​}\left(\mu_{\text{eff}}-V_{th}\right)\text{​}.$$

**Figure 3:**
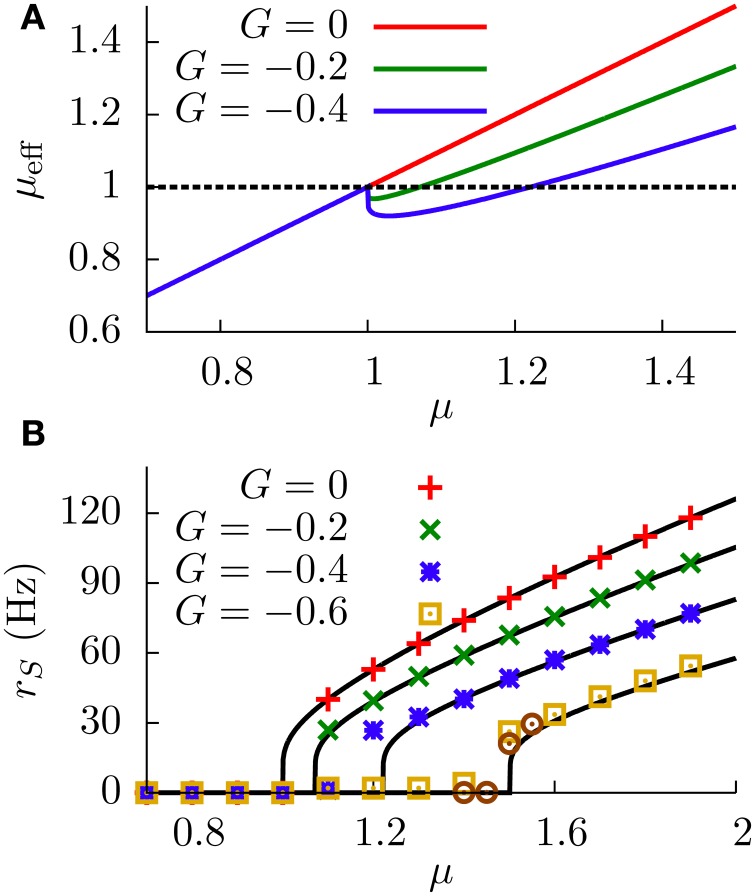
**(A)** Effective bias entering the SP neuron as a function of the sensory input μ, for the deterministic case (σ = 0). Red line (*G* = 0) indicates the effective bias for the case in which no feedforward inhibition is considered. **(B)** Response of the SP neuron to sensory input (i.e., the f-I curve of the SP cell), for the deterministic case. Numerical simulations of a network of *N* = 100 DP neurons (symbols) show a very good agreement with our theoretical estimations (lines). Circles denote the *G* = −0.6 case for a network of *N* = 500 DP neurons, indicating that the small discrepancies between simulations and theory in the firing onset are due to finite-size effects of the simulation.

The dependence of the effective bias on the sensory input, given by Equation (9), is depicted in Figure 3A. By careful inspection of this figure, one can see that the effective bias increases linearly with μ until μ = *V*_*th*_. Beyond that point, DP cells start firing and the effective bias starts decreasing, therefore frustrating the firing of the SP cells.

More precisely, the SP cells will start to fire as soon as μ_eff_ = μ + τ_*m*_*Gr*_*D*_(μ) becomes greater than *V*_*th*_. Since we have μ_eff_ < μ for *G* < 0, the onsets will occur at different μ values for SP and DP cells, leading to a shift of the gain along the μ axis. This shift becomes evident when we consider the divergences of the SP and DP gains at the firing onsets (the divergences are caused by the derivative of the Heaviside functions appearing in Equations 7, 10). For *G* < 0, the divergence of the derivative of *r*_*S*_ is shifted to the right on the μ axis with respect to the divergence of the derivative of *r*_*D*_. Such a shift of the sharp SP firing onset (with respect to the DP firing onset) constitutes a subtractive effect. In addition, for large enough values of μ, the denominator in the second term of the r.h.s. of Equation (9) tends to τ_*r*_, and the effective bias becomes then μ_eff_ ≃ μ + *G*τ_*m*_/τ_*r*_, leading again to a shift of the SP f-I curve for *G* < 0. It is worth noting that, due to the ratio of time scales τ_*m*_/τ_*r*_ ~ 10 obtained for realistic values of the parameters, μ_eff_ will be significantly smaller than μ even for moderate values of *G*, which means that SP cells would saturate much later than DP cells along the μ axis.

Based on the above reasoning, one should therefore expect to observe a subtractive effect of the feedforward inhibition on the f-I curve of the SP neuron at zero noise. This is indeed what we observe in Figure 3B, where the results of numerical simulations of the model for σ = 0 show a very good agreement with our theoretical expression given by Equation (10). We can observe that the displacement of the firing onset depends on *G* as theoretically predicted, with more negative values of *G* leading to larger shifts of the onset of firing. Therefore, the modulation of the strength *G* leads to a subtractive gain control in the f-I curve of the deterministic (σ = 0) system.

### 3.2. Divisive gain control

After analyzing the deterministic case, we can now study the more general case in which neurons in the circuit present some level of stochasticity (i.e., σ > 0) in their dynamics. Such a stochasticity may be due, for instance, to the noisy dynamics of ion channels (White et al., 2000), or to synaptic bombardment from other surrounding neurons (Hô and Destexhe, 2000), for instance (see also Longtin, 2013 for a review).

For the stochastic case σ > 0, the mean firing rate of the DP neurons may be obtained by solving Equation (1), and it is given by (Tuckwell, 1989)

(11) $$r_{D}={\left[\tau_{r}+\tau_{m}\int_{z_{r}^{D}}^{z_{th}^{D}}\sqrt{\pi }e^{z^{2}}\left(1+\text{erf}(z)\right)dz\right]}^{-1}\text{​},$$

where $z_{th}^{D}\equiv \frac{V_{th}-\mu }{\sigma }$, $z_{r}^{D}\equiv \frac{V_{r}-\mu }{\sigma }$, and erf(*z*) is the error function. The effective bias is again μ_eff_ = μ + τ_*m*_*Gr*_*D*_, although the explicit form is more complex now. Finally, we can write the mean firing rate of SP neurons as

(12) $$r_{S}={\left[\tau_{r}+\tau_{m}\int_{z_{r}^{S}}^{z_{th}^{S}}\sqrt{\pi }e^{z^{2}}\left(1+\text{erf}(z)\right)dz\right]}^{-1}\text{​},$$

where $z_{th}^{S}\equiv \frac{V_{th}-\mu_{\text{eff}}}{\sigma }$ and $z_{r}^{S}\equiv \frac{V_{r}-\mu_{\text{eff}}}{\sigma }$.

The main effect of the addition of noise to the DP neuron model is the appearance of a smooth *linearization* around the onset of the f-I curve for these neurons (Doiron et al., 2001). This is due to noise-induced firing, which is especially important when the neuron is slightly below the firing threshold.

**Figure 4:**
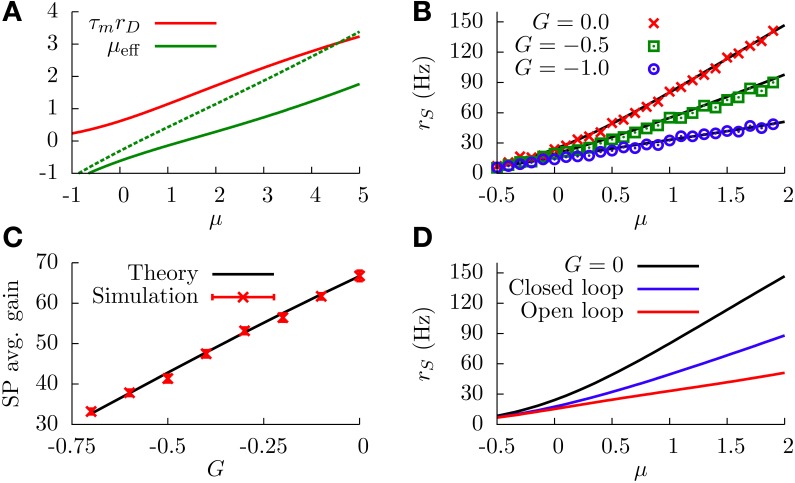
**(A)** DP firing rate (red line) and effective bias (solid green line) as a function of the sensory input μ. One can see that both curves can be considered as approximately linear with μ (this is valid as long as |*G*| is not very large). Parameters are $\sigma =\sqrt{3}$, *G* = −1. The green dotted curve is the same as the solid green line, but for *G* = −0.5. **(B)** SP firing rate as a function of the sensory input, for different values of *G* and σ = 1. The divisive gain control occurs as *G* takes larger negative values. **(C)** Average gain of the SP f-I curve as a function of *G*, for σ = 1. The average gain is obtained by fitting the f-I curve with a linear function over a range of μ values for which the curve is approximately linear. The displayed results differ by the origin of the f-I curves used. For the solid black line, the rates are computed from the analytical formula (Equation 12). For the red symbols, the f-I curves are extracted from simulations of network. The error bars come from the fitting procedure, and serve to illustrate how linear the f-I curves are for the chosen μ ranges. **(D)** Comparison between the divisive gain control in our model (open-loop feedback) and the one studied in Sutherland et al. (2009) (closed-loop feedback), for σ = 1. As the panel shows, the open-loop feedback mechanism studied here provides a stronger divisive effect than the one studied by Sutherland et al. for the same feedback strength and conditions. Both the open-loop and closed-loop cases correspond to the same strength, *G* = −1. For all panels, solid or dotted lines come from theoretical (analytical) expressions, whereas symbols come from simulations.

As a consequence of this linearization, the effective bias μ_eff_ may be approximately described, for values of μ close to the DP firing onset, as

(13) $$\mu_{\text{eff}}=\mu +\tau_{m}Gr_{D}≃\mu +\tau_{m}G\left(C_{1}\mu +C_{2}\right)\text{​},$$

where *C*_1_ and *C*_2_ are constants, with *C*_1_ » C_2_ for realistic values of the parameters. The effective bias, therefore, would be approximately linear, with a slope that depends on *G*. Such a linear dependence is shown in Figure 4A, where we can see that μ_eff_ is indeed well approximated by a linear relationship with μ, and that the slope depends on *G*. In particular, as *G* goes to more negative values the slope of the μ_eff_ − μ relationship decreases. We will restrict ourselves here to the case of *G* having relatively small absolute values, so that μ_eff_ will be an increasing function of μ in all cases. Large negative values of *G*, which could compromise this tendency, will be addressed in the next section.

Interestingly, since the f-I curve of an isolated SP neuron is also linearized by the presence of stochasticity, the above approximation can be applied again: the multiplicative effect that *G* has on the slope of μ_eff_ will also affect the slope of the SP f-I curve in the same way. Indeed, by comparison of Equations (11, 12), we can see that the response of a SP cell to μ_eff_ is identical to the response of a DP cell to μ, so we can assume the same linear dependence *r*_*S*_ ≃ *C*_1_μ_eff_ + *C*_2_ and arrive at the following divisive relationship

(14) $$r_{S}=\left(1+C_{1}\tau_{m}\;G\right)r_{D}.$$

This relationship also holds, in an approximate way, when one solves Equation (12) analytically, without considering any explicit linearization of the firing rates. This is shown in Figure 4B, where one can observe that increasing the strength of the feedforward inhibition has a divisive effect on the f-I curve of the SP neuron, as predicted. The figure also shows the good agreement between numerical simulations of the model and the theoretical expression, given by Equation (12). Figure 4C shows more clearly the relation between *G* and the SP gain, and we observe that larger negative values of *G* produce a smaller slope on the SP f-I curve. Consequently, the modulation of the feedforward inhibitory strength (in the range of small |*G*|) provides a divisive gain control to the system when stochasticity is considered.

In the mechanistic description presented above, the divisive gain modulation strongly depends on the fact that DP neurons drive the response of SP neurons without any restrictions (such as feedback from other cells to the DP neurons). This approach differs from previous attempts to explain the divisive gain control found *in vivo* (Bastian, 1986b), such as the one presented in Sutherland et al. (2009). In this previous approach, Sutherland et al. considered a unique population of ELL pyramidal cells, without distinguishing between DP and SP neurons. The system was then assumed to display a closed-loop feedback circuit, in which the population of pyramidal neurons projected their output to a feedback kernel, which in turn inhibited the activity of the population. Because the negative feedback was affecting all neurons in this case, a high firing rate would be prevented by the closed-loop inhibition. In the approach we present here, on the other hand, DP neurons (which drive the gain control of SP cells) do not receive inhibitory feedback, and therefore they are able to raise their firing rate higher and produce a stronger and more effective modulation of the SP firing rate than in the closed-loop scenario. This can be seen in Figure 4D, where our open-loop model is compared with a closed-loop version of the same model for the same values of the parameters (the case *G* = 0 is also shown for comparison). In the closed-loop model, the firing rate of the unique pyramidal cell population ν is given by

(15) $$\nu ={\left[\tau_{r}+\tau_{m}\int_{z_{r}}^{z_{th}}\sqrt{\pi }e^{z^{2}}\left(1+\text{erf}(z)\right)dz\right]}^{-1}\text{​},$$

where $z_{th}=\frac{V_{th}-\mu -\tau_{m}G\nu }{\sigma }$ and $z_{r}=\frac{V_{r}-\mu -\tau_{m}G\nu }{\sigma }$. It is worth noting that both *z*_*th*_ and *z*_*r*_ depend on the population firing rate ν, and therefore Equation (15) has to be solved recursively. As Figure 4D shows, the open-loop model proposed here allows for a stronger modulation than the closed-loop model for the same parameter values and, in particular, for the same value of *G* in both cases.

### 3.3. Non-monotonic gain control

In the previous section, we assumed small absolute values of *G* to simplify our treatment. This allowed us to understand, from a qualitative point of view, the origin of the divisive gain control in our feedforward inhibitory pathway system, and our findings were supported by both our numerical simulations and our theoretical description. However, these results could be different for larger values of *G*, as a strong feedforward inhibition driven by sensory input could overcome the excitatory effects of this same sensory input on SP cells. If that were the case, μ_eff_ would no longer increase with μ (or, at least, not for all values of μ), and the effect of *G* on the f-I curve might change. In order to explore such a possibility, we need to consider a more careful analysis of the stochastic case (i.e., σ > 0) for large values of *G*.

We start by identifying potential extrema in the f-I curve of the SP neuron, which would be an expected effect of feedforward inhibition overcoming excitation in SP cells for a given value of μ. Since the SP firing rate is a monotonically increasing function of μ_eff_ (see Equation 12), finding the extrema of the effective bias as a function of μ would be equivalent to finding the extrema of the f-I curve for SP cells. The condition for extrema of the effective bias μ_eff_ = μ + τ_*m*_*Gr*_*D*_ can be obtained from $\frac{d\mu_{\text{eff}}}{d\mu }=0$, and it gives

(16) $$\frac{dr_{D}}{d\mu }=-\frac{1}{\tau_{m}G},$$

or, in a more explicit form,

(17) $$\sigma +\sqrt{\pi }\tau_{m}^{2}Gr_{D}^{2}\left[e^{z_{th}^{2}}\left(1+\text{erf}\left(z_{th}\right)\right)-e^{z_{r}^{2}}\left(1+\text{erf}(z_{r})\right)\right]=0.$$

We will consider the condition of extrema in its simplified version (i.e., Equation 16) for clarity. The relationship *r*_*D*_ versus μ, given by Equation (11), takes a sigmoidal-like shape: *r*_*D*_ tends to zero for large negative μ, and to 1/τ_*r*_ for large positive μ. The maximum slope reached by *r*_*D*_ (which we will denote as γ) would be located somewhere between these two limits. Therefore, $\frac{dr_{D}}{d\mu }$ would follow approximately a bell shape: it is zero at both ends of the μ axis, and it has a maximum (of value γ) for a moderate value of μ.

**Figure 5:**
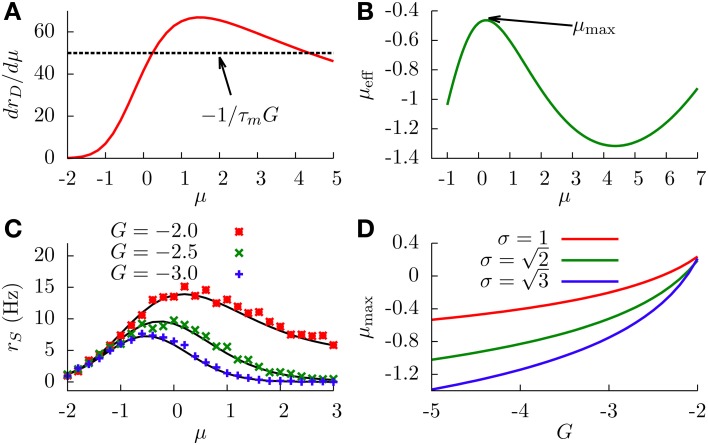
**(A)** Graphical representation of the condition for the existence of extrema (Equation 16). We can see that solutions for this equation exist only when γ (the peak value of the curve) is larger that $\frac{1}{\tau_{m}|G|}$ (dashed line). Parameters are σ = 1 and *G* = −2. **(B)** Effective bias as a function of μ, showing the existence of a maximum and a minimum. Parameters are σ = 1 and *G* = −2. **(C)** SP firing rate as a function of the input μ, for different values of *G*, displaying non-monotonic gain control. We chose $\sigma =\sqrt{2}$. **(D)** Position of the SP firing rate peak (shown in panel **C**) as a function of *G*, for different values of σ. In all panels, solid lines come from theoretical (analytical) expressions, whereas symbols come from simulations.

With this information, and together with Equation (16), it is easy to see that extrema will exist only when $\gamma >\frac{1}{\tau_{m}|G|}$, which typically occurs for large enough absolute values of the feedforward inhibitory strength. In this case, we will have two extrema (since $\frac{dr_{D}}{d\mu }$ is bell-shaped and will cross the constant level $\frac{1}{\tau_{m}\;|G|}$ twice). Figure 5A shows $\frac{dr_{D}}{d\mu }$ as a function of μ, in a situation in which the two extrema exist.

The concrete shape of the effective bias with μ is shown in Figure 5B. The two extrema correspond to a maximum (at small μ) and a minimum (at larger μ). This yields a bell-shape dependence of the effective bias on μ (at low values), followed by an increase with μ (for high values). The cause for this behavior is the following: at very small values of μ, the firing rate of the DP neurons is not high enough, and the main contribution to the effective bias is the sensory stimulus μ, with the feedforward inhibition playing a minor role. As μ makes the DP firing rate increase, the feedforward inhibition term becomes dominant (since *G* is negative and large) and the effective bias starts to decrease, completing the bell-shape profile observed in Figure 5B. After that, and for very large values of μ, the DP firing rate approaches its saturation value 1/τ_*r*_. At this point the sensory stimulus μ becomes the dominant term and μ_eff_ begins to rise again. It is worth noting that this second rising of μ_eff_ occurs at input levels where the firing rate of DP neurons saturates, and such sensory input levels are beyond the range in which biologically relevant information can be linearly processed in the system, according to electroreceptor input–output characteristics (Gussin et al., 2007). The attainment of the maximum rate, however, occurs for biologically sound biases, as exemplified in Figure 5C.

Since the firing rate of SP cells monotonically increases with the effective bias, the maximum and minimum found in μ_eff_ are also observed for the SP firing rate, as shown in Figure 5C. This implies that, for a stochastic system, enhancing the negative feedforward strength may drive the system from a divisive to a non-monotonic gain control regime. The figure also shows that the location and height of the peak of the SP firing rate is also modulated by *G*. This is to be expected, since the stronger the inhibition, the sooner the DP firing rate will start decreasing the effective bias. Large negative values of *G* will shift the location of the SP firing rate peak toward lower values of μ, as can be observed in Figure 5C and, with more detail, in Figure 5D. The level of stochasticity has also an impact on the position of the peak: larger values of σ will increase the overall firing rate of DP neurons, and the resulting increment in the feedforward inhibition will shift the peak toward even lower values of μ, as Figure 5D also shows.

**Figure 6:**
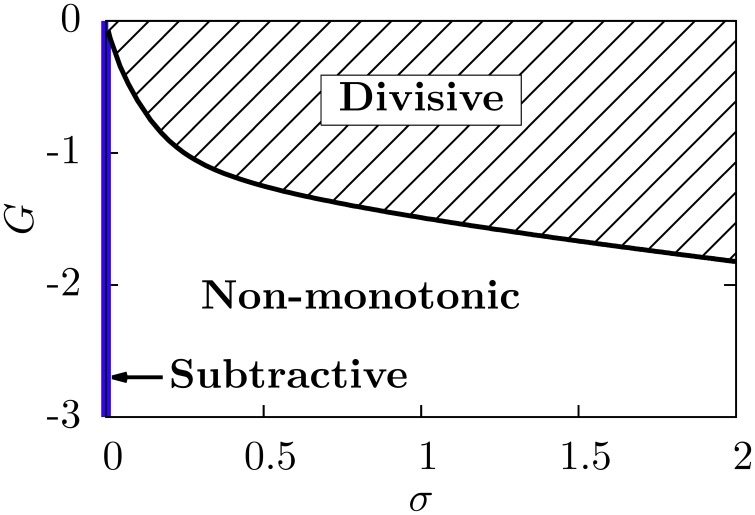
**Phase diagram of the neural system studied**. The subtractive gain control regime corresponds to the σ = 0 axis, and the critical line separating the divisive and the non-monotonic gain control regimes is given by Equation (18).

Our neural circuit, therefore, is able to display three different regimes, corresponding to subtractive, divisive, and non-monotonic gain control. As the theory and numerical simulations show, both the level of stochasticity σ and the feedforward strength *G* play an important role in the behavior of the system. Figure 6 shows a phase diagram of the system as a function of these two parameters. The subtractive gain control is observed only for deterministic systems (σ = 0), since the introduction of some level of noise would smooth the DP firing onset and we would go into other regimes. For σ > 0 the system can be in the divisive or non-monotonic regime, depending on the value of *G*. As discussed in the previous section, small absolute values of *G* would linearize the DP rate, effective bias and SP rate with respect to μ, leading to the appearance of divisive gain control. For large negative *G*, the feedforward inhibition can eventually overcome the input μ to the SP neuron and the circuit will display non-monotonic gain control. The critical line separating the divisive and non-monotonic regimes is given by

(18) $$\gamma =\frac{1}{\tau_{m}|G|}.$$

To obtain the critical line in Figure 6, we varied *G*—from within the divisive region—for each value of σ until the condition for extrema (Equation 17) at finite values of μ was first met (and actualizing Equation 18).

We emphasize that, in order to get a non-monotonicity in the SP firing rate, the derivative of the effective bias must vanish for a certain μ value, and the SP cell must have a non-zero firing rate around that value. Because of these two conditions, a non-monotonic behavior cannot be obtained for zero noise. Indeed, in the absence of noise, the DP firing rate is not differentiable at the μ value for which the maximum should occur, and furthermore the SP firing rate would be zero for that μ value.

We simplified the above analysis of the system by assuming that the SP and DP cells share the same properties, namely the same bias (μ) and the same level of noise (σ). We argue that this analysis can be extended to more general contexts. In real systems, neural attributes are not uniform. For instance, in the cortex, inhibitory interneurons fire irregularly in response to constant inputs *in vitro* (Stiefel et al., 2013), whereas pyramidal neurons are relatively regular (Mainen and Sejnowski, 1995). In the ELL, even though both the SP and the DP cells receive a common external input, they do not possess the same variability (Bastian and Nguyenkim, 2001), which may translate into different biases and noise intensities. Furthermore, even a weak neuron-to-neuron variability among the neural populations could induce non-trivial effects in information processing (Mejias and Longtin, 2012; Nicola et al., 2013). Above, we amalgamated the intrinsic bias and the applied current (*I*_app_, say) under the umbrella variable *μ*. To accommodate different neural properties, we could use two separate intrinsic biases (μ_*S*_ and μ_*D*_) and noise intensities (σ_*S*_ and σ_*D*_), and study the SP firing rate as a function of *I*_app_. This could produce slightly different results than above. As an example, for the noiseless case (σ_*S*_ = σ_*D*_ = 0), the SP cells could start to fire before the DP neurons—provided that μ_*S*_ is large enough—, stay active for a limited range of *I*_app_ values, become silent when the DP cells attain a sufficient rate, and then fire again for higher *I*_app_. This would constitute a mixed regime showing both non-monotonic and subtractive gain controls. Note that this mixed regime is also observable for uniform neural properties when the noise is low. In the latter case, the isolated blob of non-zero firing rate is due to noise.

### 3.4. Time-dependent stimuli and synaptic delays

Real neural systems deal with time-dependent stimuli. In sensory networks, neurons are responsible for encoding the temporal features of information coming from the environment. When dealing with time-dependent signals, a number of factors have to be considered in addition to the ones included up to now in our study. For instance, the existence of temporal delays in the transmission of information, due to the finite transmission speed of action potentials along the axons, becomes important. In the particular case of the ELL, signals from the DP cells have to travel through different neural populations before arriving to SP cells. Traveling such a distance causes a temporal delay in the transmission that, in some cases, may reach tens of milliseconds (Maler et al., 1991; Bol et al., 2011). Although this temporal delay did not have an effect in our previous results, with μ being constant in time (data not shown), it might have a significant impact in more realistic situations, when the sensory input presents temporal fluctuations and some level of autocorrelation. Different synaptic filter functions *s*(τ) might have an effect in these realistic situations as well.

To test our model for these conditions we use, as sensory input, a time-varying quantity $\hat{\mu }(t)$ of the form

(19) $$\hat{\mu }(t)=\mu +\zeta (t),$$

where μ is the constant bias, and ζ(*t*) is a gaussian low-pass filtered noise of zero mean and standard deviation σ_*c*_. This noise term is generated using a Butterworth fourth-order digital filter with cut-off frequency of 100 Hz. In the following, to explore the behavior of our model for other synaptic filter functions, we consider a delta-type synaptic function *s*(τ) = δ(τ − τ_*d*_).

**Figure 7:**
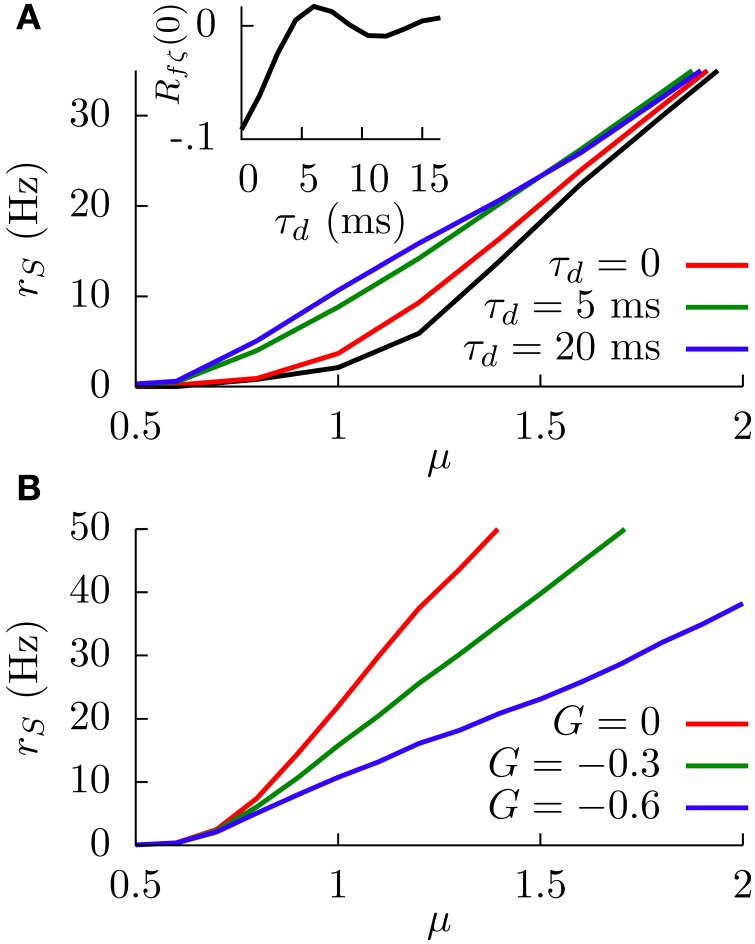
**(A)** Simulation results of the effect of the temporal delay on the f-I curve of the system at *G* = −0.6. The black line corresponds to the case in which the sensory input is just a constant bias and only the white noise is present. For other curves, the fluctuating term ζ(*t*) is also present, and the effect of varying τ_*d*_ is shown. Inset: Theoretical dependence of the covariance of *f*(*t*) and ζ(*t*) on the temporal delay. **(B)** Simulation results of the SP firing rate versus constant bias for different values of *G*, a white noise term, and a slow fluctuating term ζ(*t*), for a temporal delay τ_*d*_ = 20 ms. In both panels, we have σ = 0.1, σ_*c*_ = 0.3, and τ_*m*_ = 15 ms.

When the temporal delay τ_*d*_ is set to zero, the main effect of considering this slowly fluctuating input is a slight increase in the SP firing rate along the f-I curve, as Figure 7A shows. The black line in the figure shows the f-I curve for σ_*c*_ = 0, and the red line shows the same f-I curve for σ_*c*_ = 1. This firing rate increase is simply due to the presence of the extra fluctuating term ζ(*t*).

The figure also shows, interestingly, that considering temporal delays τ_*d*_ larger than zero leads to a significant increment in the SP firing rate, especially around the firing onset. To understand this phenomenon, it is useful to consider a slow (with respect to τ_*m*_) positive fluctuation in $\hat{\mu }(t)$, arriving at DP and SP neurons simultaneously. If the temporal delay τ_*d*_ of our circuit from DP cells to SP cells is zero, the transient increment in the sensory input received by the SP neuron will be compensated almost simultaneously (apart from the response time) by the transient increment of the DP firing rate, which will have an inhibitory effect on the SP neuron since *G* < 0. The effective bias received by the SP neuron, therefore, will be mainly unaffected by such a fluctuation.

In the presence of some level of temporal delay, however, this “compensation” from the DP cells may arrive late, maybe once the transient increment in SP firing due to the fluctuation is over. Even more, this late compensation from DP cells could cause a transient decrement in the effective bias that the SP neuron receives. As a consequence, the effective bias arriving at the SP cells will be highly fluctuating, and these input fluctuations will cause an increment in the SP firing rate. This novel variability-increasing effect would be especially important around the SP firing onset, and our simulation results confirm this point (Figure 7A). The effect of the temporal delay on the level of fluctuations in the effective bias can also be mathematically derived (see Appendix). Indeed, our calculations show (inset in Figure 7A) that the covariance of *f*(*t*) and ζ(*t*), and therefore the fluctuations of the effective bias, increases from negative values toward zero with the temporal delay τ_*d*_ (with some minor oscillatory component), supporting these findings on the delay-induced effects on SP firing rate. Time-dependent stimuli would also induce correlations between DP and SP cells, which would depend on the temporal delays along with a number of other factors (Ostojic et al., 2009).

In Figure 7B, one can see an example of the divisive gain control for this more realistic situation with delay. In this case, the sensory input has a fluctuating part ζ(*t*) and a temporal delay of 20 ms. The behavior of the system is qualitatively similar to the one observed previously (i.e., large negative values of *G* induce a divisive effect on the gain of the f-I curve). The non-monotonic behavior also holds for this more realistic model (data not shown).

In our treatment of the effect of delay on the f-I curve, we assumed that the feedforward inhibitory strength *G* is a constant. That is, *G* had the same magnitude for all frequencies contained in the signal. This may not be the case for real systems. For instance, in the weakly electric fish, the EGp parallel fibers are known to be inactive when sinusoidal signals of higher frequency than about 20 Hz are applied to the network (Bol et al., 2011). On the other hand, the direct inhibition coming from the nP seems to be active for an extended frequency range (Doiron et al., 2004). So, *G* may generally depend on frequency in a non-trivial way. This fact can easily be included into the result for the covariance of *f*(*t*) and ζ(*t*) appearing in the Appendix, provided that one knows—at least approximately—how *G* changes with frequency. If, for example, *G* is zero above a given frequency, then, obviously, signals only containing frequencies above that cutoff should have no effect on the f-I curve.

### 3.5. Comparison with experimental data

We now apply our model to *in vivo* data showing gain control in SP cells of weakly electric fish (Bastian, 1986b). In Figure 2A, reproduced from Bastian (1986b), the firing rates of SP cells in control fish is compared with those in fish for which the EGp has been lesioned. The result of this procedure is to eliminate the signal coming from the parallel fibers. Bastian's results show that the EGp contribution modulates the f-I curve of SP neurons, producing a divisive gain control. Since this form of gain control has been identified as one of the behaviors displayed by our model, it might be interesting to test whether the model is able to quantitatively explain the experimental data. Here, we shall restrict ourselves to analytical f-I curves (Equation 12) since they have been shown to be in good agreement with those extracted from numerical simulations of Equations (1, 6). The experimental results are given as relative firing rates versus the stimulus intensity in decibels. To apply our model, we first need to convert these relative firing rates to absolute ones and relate the experimental stimuli to our μ.

**Figure 8:**
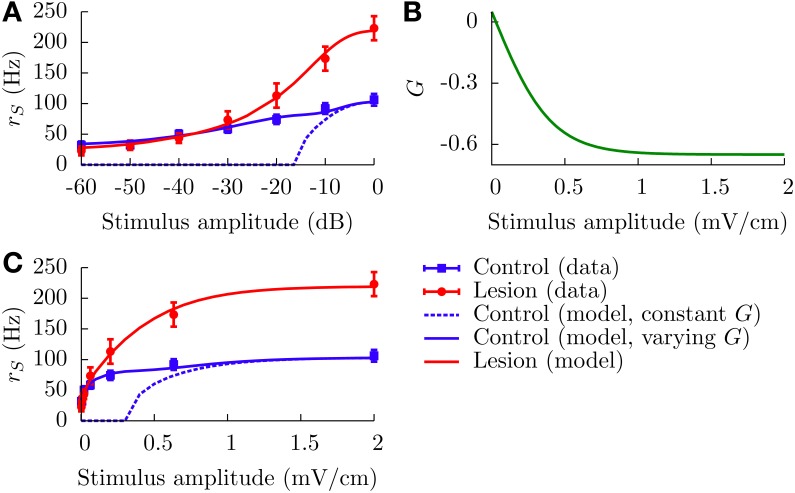
**Divisive gain control measured in SP cells *in vivo* and the theoretical predictions of our model. (A)** Absolute firing rates (in contrast to Figure 2A where rate changes relative to baseline were plotted) as a function of the stimulus amplitude in decibels. Experimental absolute rates (symbols) are obtained from the relative rates appearing in Figure 2A by adding the baseline rates for the lesion and control cases (see text for details, especially Equation 20). To simulate the lesion (i.e., absence of parallel fibers) in the model, we set *G* = 0 (red line). The theoretical control curves (solid and dashed blue lines) differ by the assumptions on *G*. For the dashed blue line, *G* = −0.65 for all amplitude modulations; for the solid one, *G* is given by the curve illustrated in panel **(B)** (*G*(*E*_AM_) = *G*_0_ + *B*/[1 + exp(−κ*E*_AM_)], with *G*_0_ = 0.75, *B* = −1.4, and κ = 5). Panel **(C)** contains the same information as panel **(A)**, but with a linear scale for the *x* axis.

The stimuli are amplitude modulations (AMs, in mV/cm) of the fish's electric field. In mathematical expressions we shall denote the intensity of these AMs by the symbol *E*_AM_. The zero-decibel reference is *E*^ref^_AM_ = 2 mV/cm (Bastian, 1986b), so that the number of decibels is given by 20 log_10_(*E*_AM_/*E*^ref^_AM_). Relative firing rates for the lesion and control cases are given by the rates for given AMs from which is subtracted the rate in the spontaneous regime (i.e., when *E*_AM_ = 0). More precisely, absolute firing rates are given by

(20) $$r_{S}^{C(L)}\left(E_{\text{AM}}\right)=r_{S}^{C(L)}(0)+\Delta r_{S}^{C(L)}\left(E_{\text{AM}}\right)\text{​},$$

where Δ*r*^*C*(*L*)^_*S*_(*E*_AM_) represents relative firing rates in the control (lesion) case. From Bastian (1986a), it appears that *r*^*C*^_*S*_(0) − *r*^*L*^_*S*_(0) ~ 10 Hz, which means that the net effect of the EGp parallel fibers is actually excitatory in the spontaneous regime. On the other hand, it is clear from Figure 2A that the EGp contribution is inhibitory for larger *E*_AM_. Therefore, this contribution changes sign somewhere between 0 and 2 mV/cm. From inspection of Figure 8A, in which absolute firing rates are plotted, this transition occurs for a small AM value (where the lesion and control curves intersect).

We now relate the AMs to our μ. The SP and DP cells receive input from electrosensory afferents, which encode the AMs of the electric field. We consider that μ encompasses an intrinsic bias μ_0_ and a simple summation of these afferent inputs. The average input to a ELL pyramidal cell is then

(21) $$\mu =\mu_{0}+\tau_{m}G_{\text{aff}}r_{\text{aff}}\left(E_{\text{AM}}\right)\equiv \mu_{0}+\gamma \left(E_{\text{AM}}\right)\text{​},$$

where *G*_aff_ is the afferent synaptic strength times the number of afferents (compare with Equation 8). The afferents' firing rate, *r*_aff_(*E*_AM_), is a sigmoid taking the form of a Boltzmann function (Benda et al., 2005):

(22) $$r_{\text{aff}}\left(E_{\text{AM}}\right)=\frac{r_{\text{max}}}{1+e^{-k\left(E_{\text{AM}}-h\right)}},$$

where *r*_max_ is the maximum firing rate. The slope of the linear part of this curve is given by *r*_max_*k*/4, whereas *h* is the AM value at which *r*_aff_ = *r*_max_/2. Replacing Equation (22) into (21) provides the required relationship between μ and *E*_AM_. We further define the constant *A* ≡ τ_*m*_*G*_aff_*r*_max_.

To fit the data corresponding to a lesioned fish, i.e., which lacks the set of parallel fibers connecting the EGp with the SP cells, we set *G* = 0. This is not rigorously true since, from Figure 2B, the direct inhibitory signal from the nP could still be active, and hence *G* should not be strictly zero. However, given the overall large effect that the parallel fibers have on the SP cells' f-I curve (Figure 2A), it seems plausible to neglect the nP component of the total feedforward inhibitory pathway in first approximation. Heuristically, we could write *G* = *G*_E*Gp*_ + *G*_*nP*_, where *G*_E*Gp*_ (respectively *G*_*nP*_) is the strength of the feedforward inhibition coming from the EGp (respectively nP). Then the effective bias would be expressed as μ_*eff*_ = μ_0,*S*_ + γ(*E*_AM_) + τ_*m*_(*G*_E*Gp*_ + *G*_*nP*_)*r*_*D*_, with μ_0,*S*_ the AM-independent bias of the SP cell, which is assumed to be different than that of the DP cells. Our approximation consists in setting μ_0,*S*_ + τ_*m*_*G*_*nP*_*r*_*D*_ ≈ μ_0,*D*_ ≡ μ_0_ and identifying *G*_E*Gp*_ with *G*.

All parameters other than *G* are assumed identical for the lesion and control cases. These parameters were determined by inspection of the lesion data and by comparison with other studies of the ELL pyramidal neurons (Doiron et al., 2003, 2004; Bol et al., 2011; Mejias et al., 2013). The time constants are τ_*m*_ = 6 ms and τ_*r*_ = 0.8 ms. The noise intensity σ is set to 0.01. Such a small value is prescribed by the overall aspect of the lesion firing rate when plotted against a linear scale for the AMs (Figure 8C). To model the input μ, we set μ_0_ = −0.16, *A* = 2.31, and *k* = 3 cm/mV. A range of plausible values for *k* has been established from an inspection of Figure 6D in Benda et al. (2005). The parameters μ_0_ and *A* are then set in such a way that the theoretical f-I curve for the lesion case adequately fits with the corresponding experimental results. Also, *h* is set to 0 mV/cm to simplify. From Figure 8A (or C), one sees that the red line (lesion) is a good fit to the experimental curve.

For the control case, making *G* a constant for the whole range of AMs is incompatible with the experimental results for low AMs. The dotted blue curves in Figures 8A,C illustrate this point. For instance, fixing *G* = −0.65 makes the theoretical f-I curve agree with the experimental results for large AMs, but it fails to model what happens for smaller AMs. The agreement for large AMs is mainly due to the saturation of the afferents' activity, i.e., μ does not vary appreciably under strong stimulations. Consequently, the divisiveness of the gain control at large amplitudes results from a different mechanism than the one presented up to now in the paper. For small AMs, given the very low noise intensity, a moderately high negative feedforward strength naturally yields a subtractive gain control. To get a divisive gain control for small AMs, we have to take into account the variation of *G* with the stimulus amplitude *E*_AM_.

As discussed above, the feedforward strength *G* changes from negative to positive values as *E*_AM_ decreases from 2 to 0 mV. The theoretical firing rate for the control case fits with the experimental one for *E*_AM_ = 2 mV if we set *G* = −0.65. It is reasonable to assume that *G* saturates for large positive and negative AM values. A simple function comprising these characteristics is a sigmoid akin to that given in Equation (22), but with a negative “*r*_max_.” It is displayed in Figure 8B for positive AMs. The solid blue curves in Figures 8A,C show a good agreement between theory and experiment when this function is used. We stress, however, that this function has been determined in a purely *ad hoc* way to illustrate that a varying *G* is necessary.

Our simple model is able to explain the divisive gain control obtained *in vivo* for the SP cells as long as the feedforward strength is allowed to change with the stimulus amplitude. As stated in section 2, the SP cells receive direct excitation and disynaptic inhibition from the parallel fibers. These contributions have different respective weights depending on the stimulus intensity, yielding a variable net feedforward strength (Lewis et al., 2007). Also, SP cells possess receptive fields of the ON center-OFF surround type (Maler, 2007). Center and surround have different activation thresholds (Bastian et al., 2002), so the proportion of inhibition and excitation depends on the intensity of AMs. Such a differential activation might have an impact on phenomena depending on a neuron's activity, namely the synaptic plasticity.

## 4. Discussion

The modulation of input–output characteristics is a major strategy used by neural systems for the processing and gating of information. Neurons able to change their f-I (firing rate versus input) curve stand as a prominent example of this. While very general mechanisms are known to produce subtractive gain control, mechanisms providing other forms of modulation, such as divisive or non-monotonic gain control (see Figure 1), have been harder to identify (Holt and Koch, 1997; Chance and Abbott, 2000; Doiron et al., 2001; Chance et al., 2002; Longtin et al., 2002; Sutherland et al., 2009). In this work, we presented a biologically plausible mechanism which yields subtractive, divisive and non-monotonic gain control. We analyzed, both with theoretical approaches and numerical simulations, the conditions for which each behavior may appear. The mechanism is based on the interaction between noise and some level of input-driven feedforward inhibition, and constitutes an example of a generic neural strategy which can produce different types of computationally-desirable modulations of f-I curves.

The concrete neural circuit considered in the study is very general: a feedforward neural assembly inhibits target neurons, and both populations receive a common external input. Such a circuit has already been used to study the role of feedforward inhibition in decorrelating neural activity in the somatosensory cortex of rats (Middleton et al., 2012). In the present work, it is used to model the ELL of the weakly electric fish. The feedforward population is composed of so-called deep pyramidal (DP) neurons, whose targets are neurons that inhibit superficial pyramidal (SP) cells. It is the modulation of the SP cells' firing activity that is studied here. However, the simplicity of the network and neuron models suggests that our results could apply to other systems with a similar structure.

As stated before, the f-I behavior of the neural network depends on the level of neural noise and the strength of the feedforward inhibition. In most situations, when neurons present some level of stochasticity, the feedforward inhibitory strength will determine whether the gain control is divisive or non-monotonic. This makes the system highly versatile, since the feedforward inhibitory strength, which can be seen as efficacies of the synaptic connections involved, could be modulated by long-term synaptic plasticity. This might be the case in the ELL of the weakly electric fish, as different types of long-term plasticity have been identified in the parallel fiber synapses with SP neurons in this and other species of electric fish (Han et al., 2000; Harvey-Girard et al., 2010). If that were the case, then as the inhibitory feedforward contribution becomes stronger, the divisive gain control observed experimentally in SP cells would turn into a non-monotonic gain control. Indeed, non-monotonic f-I curves for SP neurons have been recently observed *in vitro* under direct parallel fiber stimulation (Figure 3C in Lewis et al., 2007), supporting this hypothesis. Further experimental work, however, is needed to adequately test our prediction of non-monotonic gain control emerging for strong feedforward inhibition.

The existence of a non-monotonic gain control regime, as hypothesized by our modeling study and suggested by recent experimental findings (Lewis et al., 2007), would have interesting functional advantages with respect to monotonic relationships. For instance, a non-monotonic dependence in the f-I curve of a neural circuit would allow for having a high gain (i.e., high sensitivity) for low and moderate input levels while ensuring, by decreasing firing rates, that the energy constraints of the circuit are met for large inputs (something that a monotonic f-I curve cannot provide). Interestingly, it has been proposed that the non-monotonic input–output relationship caused by a combination of short-term synaptic depression and facilitation could be important for detection of coincident spikes (Mejias and Torres, 2008) and burst transmission (Izhikevich et al., 2003). Similar features might be present in our system as well, since the non-monotonic input–output profiles that we obtained via feedforward inhibition are similar to those given by short-term synaptic plasticity.

The gain control mechanism presented here holds for more realistic situations, such as when considering a time-dependent sensory stimulus. In the presence of complex input signals, the presence of temporal delays in the systems becomes an important factor, and we have considered it in our study as well. The introduction of a temporal delay in our feedforward inhibitory term revealed a novel effect in the dynamics of the system: as the delay increases, an excitatory input fluctuation arriving to a SP cell from the senses does not coincide in time with its inhibitory counterpart arriving from the inhibitory feedforward pathway. As a consequence, the level of fluctuations arriving at SP cells increases, which in turn increases the SP firing rate, especially near the firing onset. Our theoretical approach is able to explain this effect as well (see Appendix). In spite of these new effects in the dynamics of SP cells, the feedforward-driven gain control remains the same as in the original model, indicating that the mechanism can work as well for time-dependent stimuli.

Finally, we found that the basic model used to obtain the aforementioned gain control regimes is able to describe the divisive gain control observed *in vivo* in the SP cells of the ELL (Bastian, 1986b) for moderate to large stimuli. For low amplitude stimuli, the control has a significant subtractive component. Once the model is augmented with a feedforward inhibitory strength that depends on stimulus intensity, it exhibits divisive behavior at all amplitudes (i.e., even for low amplitude stimuli). This illustrates the versatility of our model, and shows how it can be modified to depict more subtle gain control phenomena in real systems.

### Conflict of interest statement

The authors declare that the research was conducted in the absence of any commercial or financial relationships that could be construed as a potential conflict of interest.

## A. Appendix

In this Appendix, we consider the situation where the SP cell receives the external input μ + ζ(*t*) (cf. Equation 19), together with the delayed synaptic inputs from the DP cells, *f*(*t*). Since ζ(*t*) is zero-mean, the SP cell receives on average a signal μ + τ_*m*_*GrD*. The goal here is to determine the intensity of the fluctuations of the total input with respect to that average. These fluctuations play the role of an effective noise which alters the f-I curve of the SP cell. In particular, we determine the effect of the delay on the magnitude of the fluctuations.

We shall compute the auto-covariance function of *f*(*t*) + ζ(*t*)—we discard the constant μ—, defined by

(23) $$\begin{array}{l} C_{f+\zeta }\left(\tau \right)\equiv \langle \left[f(t)+\zeta (t)\right]\left[f\left(t+\tau \right)+\zeta \left(t+\tau \right)\right]\rangle -\tau_{m}^{2}G^{2}r_{D}^{2}\\ \;=C_{f}\left(\tau \right)+R_{f\zeta }\left(\tau \right)+R_{f\zeta }\left(-\tau \right)+R_{\zeta }\left(\tau \right)\text{​}, \end{array}$$

where *C*_*f*_(*t*) ≡ 〈*f*(*t*)*f*(*t* + τ)〉 − τ^2^_*m*_*G*^2^*r*^2^_*D*_ is the auto-covariance function of *f*(*t*), *R*_*f*ζ_(τ) ≡ 〈*f*(*t*)ζ(*t* + τ)〉 is the cross-correlation function of *f*(*t*) and ζ(*t*), and *R*_ζ_(τ) ≡ 〈ζ(*t*)ζ(*t* + τ)〉 is the auto-correlation function of ζ(*t*). The brackets 〈·〉 signify averages over realizations of the internal (ξ^*D*^_*i*_) and external (ζ) noises. We compute these quantities separately below.

### A.1 Auto-covariance of *f(t)*

We consider both delta and alpha synapses. We first get the power spectrum of *f*(*t*) and then Fourier transform it to get the auto-correlation function, from which we subtract τ^2^_*m*_*G*^2^*r*^2^_*D*_ to get the auto-covariance function. Consider first the following restriction of all *y*^*D*^_*i*_ to the interval [0, *T*], i.e.,

$$y_{i,T}^{D}\equiv \{\begin{array}{c} y_{i}^{D}(t)\text{ for }t\in [0,T]\\ 0\text{ elsewhere} \end{array}\text{​​}.$$

We define the Fourier operator, , as

and its inverse, , as

With $f_{T}(t)\equiv \tau_{m}\frac{G}{N}\sum_{i}\left(s*y_{i,T}^{D})(t\right)$, we have

The power spectrum of *f*(*t*) is defined as

$$S_{f}\left(\omega \right)=\underset{T\to \infty }{\lim}\frac{\langle |{\tilde{f}}_{T}\left(\omega \right){|}^{2}\rangle }{T}.$$

Using Equation (24), we get

$$S_{f}\left(\omega \right)=\tau_{m}^{2}\frac{G^{2}}{N^{2}}|\tilde{s}\left(\omega \right){|}^{2}\sum_{i,j}\underset{T\to \infty }{\lim}\frac{\langle {\tilde{y}}_{i,T}^{D}\left(\omega \right){\tilde{y}}_{j,T}^{D^{*}}\left(\omega \right)\rangle }{T}.$$

Analogously to Equation (25), we define the cross-spectrum of two DP cells as

(26) $$S_{D}^{\text{cross}}\left(\omega \right)=\underset{T\to \infty }{\lim}\frac{\langle {\tilde{y}}_{i,T}^{D}\left(\omega \right){\tilde{y}}_{j,T}^{D^{*}}\left(\omega \right)\rangle }{T}\;\left(i\neq j\right)\text{​}.$$

Given the homogeneity of the deep cell layer, all DP cells share the same power spectrum, *S*_*D*_(ω), and all cross-spectra are the same for any neuron pairs. Hence,

(27) $$S_{f}(\omega )=\tau_{m}^{2}\frac{G^{2}}{N}|\tilde{s}(\omega ){|}^{2}\left[S_{D}(\omega )+(N-1)S_{D}^{\text{cross}}(\omega )\right]\text{​}.$$

One may interpret $|\tilde{s}(\omega ){|}^{2}$ as the energy spectrum of the synaptic filter. For *N* = 1, we get the usual output power spectrum of a linear system with impulse response function τ_*m*_*Gs*(*t*).

If we assume that DP cells' spike trains are wide-sense stationary random processes, it should be the case for *f*(*t*) as well. By the Wiener–Khintchin theorem, the auto-correlation function of *f*(*t*) is then

Since

where *s*(*t*) ≡ *s*(−*t*), we get

$$R_{f}(\tau )=\tau_{m}^{2}\frac{G^{2}}{N}\{\left(\underline{s}*s\right)*\left[R_{D}+(N-1)R_{D}^{\text{cross}}\right]\}(\tau ).$$

On the one hand, for α synapses,

$$\int_{-\infty }^{\infty }\left(\underline{s}*s\right)(t)dt={\left(\int_{-\infty }^{\infty }s(t)dt\right)}^{2}=1.$$

On the other hand, for δ synapses, $|\tilde{s}(\omega ){|}^{2}=1$, so that

Therefore, we can write

$$C_{f}(\tau )=\tau_{m}^{2}\frac{G^{2}}{N}\{\left(\underline{s}*s\right)*\left[R_{D}+(N-1)R_{D}^{\text{cross}}-Nr_{D}^{2}\right]\}(\tau ),$$

or

(29) $$C_{f}(\tau )=\tau_{m}^{2}G^{2}\left\{\left(\underline{s}*s\right)*\left[\frac{R_{D}-R_{D}^{\text{cross}}}{N}+C_{D}^{\text{cross}}\right]\right\}(\tau ).$$

For α synapses,

(30) $$\begin{array}{l} \left(\underline{s}*s\right)(t)=\frac{1}{4\tau_{s}}[\left(1+\frac{t}{\tau_{s}}\right)e^{-\frac{t}{\tau_{s}}}\Theta (t)+\\ \;\left(1-\frac{t}{\tau_{s}}\right)e^{\frac{t}{\tau_{s}}}\Theta (-t)]\text{​}. \end{array}$$

For δ synapses, (*s* ∗ *s*)(*t*) = δ(*t*) from Equation (28), hence

(31) $$C_{f}(\tau )=\tau_{m}^{2}G^{2}\left[\frac{R_{D}(\tau )-R_{D}^{\text{cross}}(\tau )}{N}+C_{D}^{\text{cross}}(\tau )\right].$$

Since *R*_*D*_(τ) contains a Dirac delta function, δ(τ), there is a white noise component due to the synaptic bombardment in the case of δ synapses. This component is low-pass filtered in the case of α synapses.

### A.2 Autocorrelation of ζ

Given that ζ is a zero-mean, low-pass filtered Gaussian white noise of standard deviation σ_*c*_, its auto-correlation function is

(32) $$R_{\zeta }(\tau )=\sigma_{c}^{2}\frac{\sin\left(\omega_{c}\tau \right)}{\omega_{c}\tau },$$

where ω_*c*_ = 2π*f*_*c*_, with *f*_*c*_ being the cutoff frequency.

### A.3 Cross-correlation function (*R*_*f*ζ_(τ) + *R*_*f*ζ_(−τ))

We now compute *R*_*f*ζ_(τ) + *R*_*f*ζ_(−τ) in Equation (23). We will conclude that this term only depends on the delay τ_*d*_. We will then obtain an expression for the covariance *R*_*f*ζ_(0), which represents the intensity of the effective noise caused by the interaction of *f* and ζ. This noise is responsible for the linearization of the f-I curve observed in Figure 7A.

Since *S*_*f*ζ_(−ω) = *S*^*^_*f*ζ_(ω), we have

where

(34) $$S_{f\zeta }(\omega )=\underset{T\to \infty }{\lim}\frac{\langle {\tilde{f}}_{T}\left(\omega \right){\tilde{\zeta }}_{T}^{*}\left(\omega \right)\rangle }{T}.$$

Using Equation (24), we get

(35) $$\begin{array}{l} S_{f\zeta }(\omega )=\tau_{m}\frac{G}{N}\tilde{s}(\omega )\sum_{i}\underset{T\to \infty }{\lim}\frac{\langle {\tilde{y}}_{i,T}^{D}(\omega ){\tilde{\zeta }}_{T}^{*}(\omega )\rangle }{T}\\ \;=\tau_{m}G\tilde{s}(\omega )S_{D\zeta }(\omega ), \end{array}$$

where again we have used the homogeneity of the DP cells. *S*_*D*ζ_(ω) is the cross-spectrum of any DP cell with the external input. Provided that the variance of ζ is not too large, we can write

(36) $${\langle {\tilde{y}}_{i,T}^{D}\left(\omega \right)\rangle }_{|\zeta_{T}}={\tilde{A}}_{\mu ,\sigma }^{D}(\omega ){\tilde{\zeta }}_{T}(\omega )+2\pi r_{D}\delta (\omega ),$$

where the notation 〈*x*〉_|ζ_*T*__ means the average of *x* for a given sample function restricted to [0, *T*] of the process ζ (“frozen noise”), taken over realizations of the internal noise. In the above equation, *r*_*D*_ is the rate evaluated with Equation (11) without the time-dependent external input ζ(*t*). The susceptibility (or transfer function) Ã^*D*^_μ,σ_(ω) depends on μ, σ, and τ_*r*_ (Lindner et al., 2005):

with Δ = [*V*^2^_*r*_ − *V*^2^_*th*_ + 2μ(*V*_*th*_ − *V*_*r*_)/2σ^2^, and  are parabolic cylinder functions. Multiplying both sides of Equation (36) by ${\tilde{\zeta }}_{T}^{*}(\omega )$, averaging over realizations of ζ_*T*_, dividing by *T* and taking the limit *T* → ∞ gives

(37) $$S_{D\zeta }(\omega )={\tilde{A}}_{\mu ,\sigma }^{D}(\omega )S_{\zeta }(\omega ),$$

where *S*_ζ_(ω) is the power spectrum of ζ. Substituting this into Equation (35) and using Equation (33) yields

Since *S*_ζ_(ω) = (σ^2^_*c*_/2*f*_*c*_)[Θ (ω + ω_*c*_) − Θ(ω − ω_*c*_)], we obtain

(39) $$\begin{array}{l} R_{f\zeta }(\tau )+R_{f\zeta }(-\tau )=\frac{\tau_{m}G\sigma_{c}^{2}}{f_{c}}\int_{\text{-}\omega_{\text{c}}}^{\omega_{\text{c}}}Re\left[\tilde{s}(\omega ){\tilde{A}}_{\mu ,\sigma }^{D}(\omega )\right]\times \\ \;e^{-i\omega \tau }\frac{d\omega }{2\pi }. \end{array}$$

Of all the contributions to *C*_*f*+ζ_(τ), only *R*_*f*ζ_(τ) + *R*_*f*ζ_(−τ) depends on the delay τ_*d*_ (through the Fourier transform of *s*(*t*), which makes *e*^*i*ωτ_*d*_^ appear). In the inset of Figure 7A we display the covariance of *f* and ζ, *R*_*f*ζ_(0), for delta synapses. Writing Ã^*D*^_μ,σ_(ω) = |Ã^*D*^_μ,σ_(ω)|*e*^*i*ϕ_*A*_(ω)^ and $\tilde{s}(\omega )=e^{i\phi_{s}}$, with ϕ_*s*_ = ωτ_*d*_, we get, from Equation (39),

(40) $$R_{f\zeta }(0)=\frac{\tau_{m}G\sigma_{c}^{2}}{2\omega_{c}}\int_{-\omega_{c}}^{\omega_{c}}|{\tilde{A}}_{\mu ,\sigma }^{D}(\omega )|\cos\left[\phi_{A}(\omega )+\phi_{s}(\omega )\right]\text{​}d\omega .\;$$

The expression above can be calculated with Maple. A LIF neuron model can be seen as a low-pass filter. Precisely, |Ã^*D*^_μ,σ_(ω)| is an even function which decreases to zero for large ω values. For a small ω_*c*_, ϕ_*A*_ ≈ βω on [−ω_*c*_, ω_*c*_], with β a positive proportionality constant, so that

(41) $$R_{f\zeta }(0)\approx \frac{\tau_{m}G\sigma_{c}^{2}}{2\omega_{c}}\int_{-\omega_{c}}^{\omega_{c}}|{\tilde{A}}_{\mu ,\sigma }^{D}(\omega )|\cos\left[\left(\beta +\tau_{d}\right)\omega \right]d\omega .$$

For τ_*d*_ < −β + π/2ω_*c*_, the first zero-crossing of cos[(β + τ_*d*_)ω] lies outside the interval [−ω_*c*_, ω_*c*_]. When τ_*d*_ increases from 0 to −β + π/2ω_*c*_, *R*_*f*ζ_(0) increases monotonically with τ_*d*_ (because *G* < 0) This increase continues until the second zero-crossing of cos[(β + τ_*d*_)ω] reaches ±ω_*c*_. Hence, a maximum occurs at τ_*d*_ = −β + 3π/(2ω_*c*_). Beyond that value, *R*_*f*ζ_(0) oscillates as more and more of the function cos[(β + τ_*d*_)ω] gets compressed into [−ω_*c*_, ω_*c*_]. For very large delays, the integral tends to zero. In summary, this calculation shows that the noise component coming from the delayed interaction of the feedforward function *f*(*t*) with the time-varying input ζ(*t*) increases monotonically for small delays. For larger delays, it starts to show damped oscillations around zero (see inset of Figure 7A).
